# Controllable Synthesis of Sheet-Flower ZnO for Low Temperature NO_2_ Sensor

**DOI:** 10.3390/nano13081413

**Published:** 2023-04-19

**Authors:** Mingjia Bai, Chaoyang Li, Xiaojun Zhao, Qingji Wang, Qinhe Pan

**Affiliations:** 1 State Key Laboratory of Marine Resource Utilization in South China Sea, College of Information and Communication Engineering, Hainan University, Haikou 570228, China; 2 School of Chemical Engineering and Technology, Hainan University, Haikou 570228, China

**Keywords:** water bath, controllable synthesis, NO_2_, gas sensor

## Abstract

ZnO is a wide band gap semiconductor metal oxide that not only has excellent electrical properties but also shows excellent gas-sensitive properties and is a promising material for the development of NO_2_ sensors. However, the current ZnO-based gas sensors usually operate at high temperatures, which greatly increases the energy consumption of the sensors and is not conducive to practical applications. Therefore, there is a need to improve the gas sensitivity and practicality of ZnO-based gas sensors. In this study, three-dimensional sheet-flower ZnO was successfully synthesized at 60 °C by a simple water bath method and modulated by different malic acid concentrations. The phase formation, surface morphology, and elemental composition of the prepared samples were studied by various characterization techniques. The gas sensor based on sheet-flower ZnO has a high response value to NO_2_ without any modification. The optimal operating temperature is 125 °C, and the response value to 1 ppm NO_2_ is 125. At the same time, the sensor also has a lower detection limit (100 ppb), good selectivity, and good stability, showing excellent sensing performance. In the future, water bath-based methods are expected to prepare other metal oxide materials with unique structures.

## 1. Introduction

Since the new century there has been rapid social and economic development, especially in recent years, and as the process of urbanization has accelerated people’s ownership of motor vehicles has increased, leading to the increasing emissions of pollutants such as nitrogen oxides in the environment; thus, air pollution has become an urgent environmental problem at this stage in the country. As a major component of nitrogen oxides, NO_2_ poses a great danger to the environment and people’s health. NO_2_ leads to the destruction of the atmospheric ozone layer and contributes to the greenhouse effect, and NO_2_ is an important cause of acid rain, photochemical smog pollution, and atmospheric haze in cities [1]. At the same time, nitrogen dioxide can enter the human body with people’s breathing, so the increase in nitrogen dioxide content in the daily environment will lead to more nitrogen dioxide entering the human body. When nitrogen dioxide inhalation occurs in excess, it will stimulate the respiratory mucosa and damage the human lungs, leading to neurological weakness and many other hazards; where exposure is above 1ppm, NO_2_ can cause corrosive effects on human eyes and lung cavities. In addition, especially for people with weak resistance such as children, the elderly, and people suffering from respiratory diseases, the impact is especially prominent [2,3]. At present, the equipment for detecting NO_2_ in the environment is large, expensive, and complicated to operate, which cannot meet the detection needs in the new situation. The semiconductor metal oxide gas sensor can achieve effective detection of NO_2_ in the environment because of its small size, low cost, and simple operation [4,5]. However, semiconductor metal oxide gas sensors still suffer from low sensitivity and high operating temperatures. Therefore, it is urgent to develop a NO_2_ gas sensor with a low detection limit, low operating temperature, and high response value.

In recent decades, gas sensors based on metal oxide semiconductors (MOSs) have been widely used due to their significant advantages, such as low cost, simple fabrication, and high compatibility with modern electronic devices [6,7]. Various MOS materials have been extensively investigated as potential gas-sensing materials, which have shown promising applications, such as SnO_2_, ZnO, In_2_O_3_, and WO_3_ [8,9,10]. However, with the continuous development of society, the requirements for gas sensors are increasing. Most MOS gas sensors cannot meet the current needs due to their poor selectivity, high detection limit, and high working temperature. It is well known that sensor performance is closely related to the morphology of the gas-sensing material [11]. In recent years, researchers have been seriously trying to solve the designs of morphology and controllable strategies of synthesis in order to improve the performances of MOS-based gas sensors [12]. Different micro/nanostructures such as nanoparticles [13], nanorods [14], nanosheets [15], hollow microspheres [16], nanoflowers, and some complex hierarchical structures [17] have been synthesized and studied as gas sensors.

As an important broadband semiconductor with unique electrical, optical, piezoelectric, and other physical properties, ZnO has very wide application prospects in the fields of microelectronic devices, photonic devices, acoustic surface wave devices, field emission devices, etc. Zinc oxide is widely used in the field of gas sensing for the detection of toxic and hazardous gases. First, ZnO is an n-type II-VI semiconductor with a wide band width (3.37 eV), large exciton binding energy (60 meV), and high electron mobility (0.022 m^2^/(V-s)). In the general environment, ZnO exhibits a non-centrosymmetric stable structure of hexagonal fibrous zincite [18]. In addition, ZnO has the advantages of good biocompatibility and chemical stability, being friendly to the environment, and a low cost of synthesis, so it has become a current research hot spot for the detection of toxic and harmful gases. It is worth mentioning that ZnO-based sensors are surface-controlled, and the sensing performance depends largely on the morphology, porosity, and crystal structure [19,20,21]. With the continuous development in related research, ZnO with complex morphologies have attracted extensive research interest in recent years, especially ZnO with three-dimensional nanostructures. For example, Yu et al. successfully prepared a 3D lettuce-like ZnO gas sensor by a one-step hydrothermal method, which has a high response value (113.04 for 100 ppm H_2_S) and selectivity for H_2_S [22]. R. Sankar Ganesh et al. prepared a 3D flower-like structure-based Ag/ZnO gas sensor, which exhibited good selectivity with a high response value (29.5% for 100 ppm ammonia) for ammonia [23]. Srijita Nundy et al. prepared a flower-like ZnO gas sensor based on the hydrothermal method, which has a high response value (28.68 for 0.74 ppm NOx) and good selectivity for NOx at room temperature [24]. Chen et al. prepared an Fe-doped ZnO nanosheet flower gas sensor by the hydrothermal method, which has a high response value (105.7 for 100 ppm acetone) and low lower detection limit (the 1 ppm acetone response value was 2.36) for acetone [25]. Therefore, it is important to develop a facile and environmentally friendly method to synthesize 3D layered ZnO nanostructures with controlled morphology. As far as we know, the existing methods for preparing ZnO with three-dimensional nanostructures include the hydrothermal method, solvothermal method, chemical co-precipitation method, and sol-gel methods. Among them, the hydrothermal method is the most commonly used method but this method has high temperatures and high cost. Therefore, it remains a great challenge to fabricate 3D ZnO for gas sensors in a facile, low-temperature, and economical method.

In this paper, three-dimensional sheet-flower ZnO hierarchical structures with controlled morphology were successfully prepared by a simple water bath method, the prepared samples were then sintered to improve the stability of the material. The synthesized sheet-flower ZnO was characterized by XRD and SEM images. At the same time, the gas-sensing characteristics of the sensor were tested, and the results showed that the sensors had good selectivity and stability. Notably, the sheet-flower ZnO hierarchical structure showed higher response values to NO_2_ gas at a lower operating temperature (125 °C), which has good application prospects.

## 2. Experimental Methods

### 2.1. Chemicals

Zinc nitrate hexahydrate (Zn(NO_3_)·6H_2_O, 99%), hexamethylene tetramine (C_6_H_12_N_4_, 99%), L-(-)-Malic acid (C_4_H_5_O_6_, 98%), isopropyl alcohol (C_3_H_8_O, 99.7%), and sodium hydroxide (NaOH, 97%) were purchased from Aladdin. All reagents used were of analytic purity and required no further purification.

### 2.2. Synthesis of ZnO Nanosheet Flowers

In a typical procedure, 2 mmol of hexamethylene tetramine was added to 50 mL of deionized water, then 4 mmol of Zinc nitrate hexahydrate and 15 mmol of L-(-)-Malic acid were added to the hexamethylene tetramine solution, and then 10 mL of isopropanol was added to the mixed solution with continuous stirring for five minutes. The prepared solution was put into a water bath at 60 °C and continuously stirred for 25 min, followed by the addition of 20 mL NaOH (2.5 M in water) and continued stirring for 5 min, and then the precipitate was separated by centrifugation and washed several times using deionized water and anhydrous ethanol in turn to remove impurities. Finally, the obtained sample was dried in a desiccator at 80 °C for 12 h. Then, it was heated up to 450 °C at 5 °C/min in a muffle furnace and kept for 2 h to obtain ZnO nanosheet flowers. At the same time, two ZnO materials were prepared with 11 mmol and 18 mmol malic acid under the same experimental conditions.

### 2.3. Characterization

The morphological properties of the sensing material were characterized by SEM (SEM Quanta 400 FEG, Eindhoven, The Netherlands). To perform transmission analysis of the prepared samples, we used transmission electron microscopy (TEM, FEI G2F20, Brno, Czech Republic). In order to determine the crystalline phase of the prepared samples, we performed XRD analysis, and X-ray diffraction (XRD) test was performed using a Rigaku MiniFlex 600, Tokyo, Japan (Cu Kα1 radiation, λ = 1.5406 Å) operated at 40 kV, 15 mA.

### 2.4. Fabrication and Measurement of Gas Sensors

**Figure 1:**
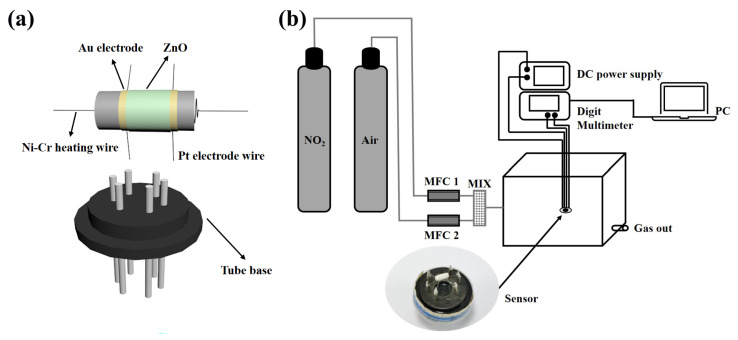
Schematic illustrations of (**a**) the gas sensor, and (**b**) the gas-sensing test system.

The gas sensors were fabricated as follows. The prepared ZnO powder was mixed with deionized water to form a paste, which was coated on an Al_2_O_3_ ceramic tube having a pair of gold electrodes and four Pt wires, as shown in Figure 1a. Then, the ceramic tube was dried at 80 °C and annealed at 400 °C for 2 h. A Ni–Cr alloy coil is passed through the ceramic tube as a heating line to adjust the operating temperature of the sensor. Finally, the ceramic tube is soldered to the hexagonal tube holder and then connected to the test line for gas sensitivity testing.

All gas sensitivity tests are done in the ultra-clean room of the laboratory, which is kept at a constant temperature of 25 °C and a constant humidity of 50%. The instrumentation and electronic circuits used for the gas sensitivity test are shown in Figure 1b. It mainly includes a DC power supply, a multifunction multimeter, and a computer. Among them, the DC power supply mainly provides a constant current to the Ni–Cr alloy coil to provide the working temperature of the sensor and the multi-function multimeter is mainly used to collect the resistance change of the sensor and display the data in real-time on the computer. The sensor was aged at 300 °C for 24 h to improve the stability of the sensor before conducting the gas sensitivity test. When the gas to be measured is an oxidizing gas, the gas response is defined as S = Rg/Ra, where Rg and Ra are the resistance of the sensor in the gas and air to be measured. The response time is defined as the time required for the sensor to reach 90% of the equilibrium value of the change in resistance in the gas to be measured, and the recovery time is defined as the time necessary for the sensor to return to 10% above the original conductance in air after releasing the test gas.

## 3. Results and Discussion

### 3.1. Structural and Morphological Characteristics

**Figure 2:**
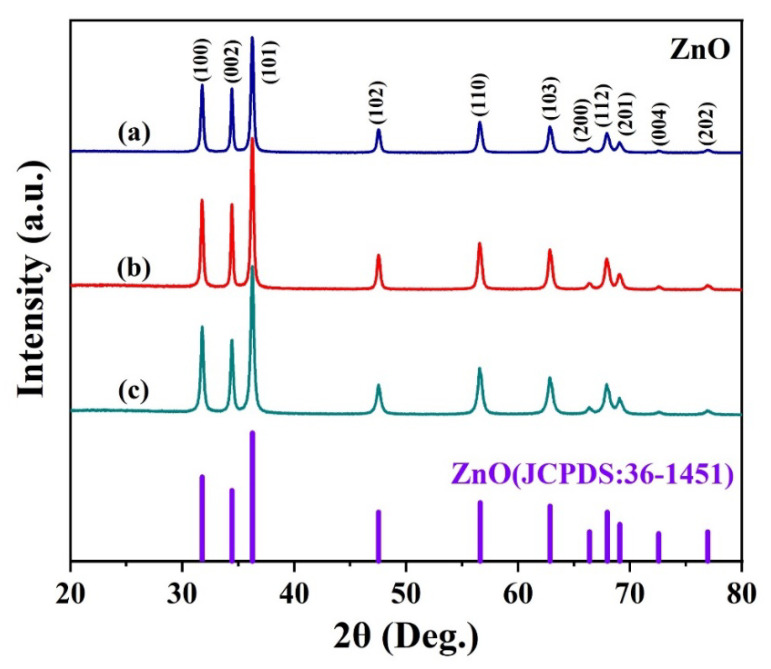
XRD patterns of ZnO nanosheet flowers assisted by different concentrations of malic acid: (**a**) 11 mmol, (**b**) 15 mmol, (**c**) 18 mmol.

The crystalline phase of the prepared sample can be obtained by XRD, the XRD patterns of ZnO prepared with different concentrations of malic acid are shown in Figure 2. As can be seen from the figure, the data of all three samples matches well-crystallized with a crystal structure of wurtzite ZnO (JCPDS card No. 36-1451) indicating that the crystal structures of the three samples are consistent [26]. In the XRD pattern, the diffraction peaks of the three samples at 31.77, 34.42, 36.25, 47.54, 56.6, 62.86, 66.38, 67.96, 69.09, 72.56, and 76.95 are corresponding to the (100), (002), (101), (102), (110), (103), (200), (112), (201), (004), (202) planes of wurtzite ZnO, respectively. In addition, no other characteristic impurity peaks were observed in the XRD patterns of the three samples, and no different crystal structures existed, further indicating that the synthesized products were all ZnO.

**Figure 3:**
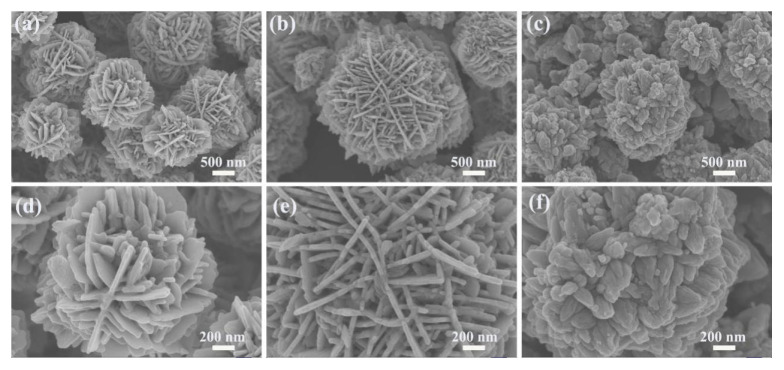
SEM images of ZnO nanosheet flowers assisted by different concentrations of malic acid: (**a**,**d**) 11 mmol; (**b**,**e**) 15 mmol; (**c**,**f**) 18 mmol.

The surface morphology of the samples was investigated by SEM. The SEM images of ZnO produced with 11 mmol malic acid are shown in Figure 3a,d. As can be seen in the figure, the sample consists of ZnO flower-like microspheres with diameters between 1–2 μm and no other morphology. The high magnification SEM of Figure 3d shows that the ZnO flower-like microspheres are composed of two-dimensional nanosheets with a thickness of about 30 nm. The SEM images of ZnO produced with 15 mmol malic acid are shown in Figure 3b,e. As can be seen in the figure, the sample consists of sheet-flower ZnO with diameters ranging from 2–3 μm. The high magnification SEM of Figure 3e shows that the microspheres have a three-dimensional layered structure and are assembled from plate-like nanosheets. The thickness of these nanosheets is about 30–40 nm, forming a network-like surface of the microspheres. In addition, a large number of nanosheets are alternately connected to each other, generating many pores. The SEM images of ZnO produced with 18 mmol malic acid are shown in Figure 3c,f. It can be observed that the ZnO microspheres are assembled from nanorods with an average size of 2–3 μm.

**Figure 4:**
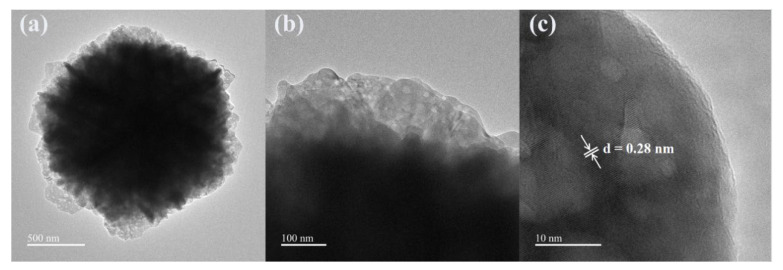
TEM and HRTEM images of the ZnO nanosheet flowers prepared at a malic acid concentration of 15 mmol: (**a**) low- and (**b**) high-magnification TEM images, and (**c**) HRTEM image.

By changing the concentration of malic acid, we are able to control the crystal morphology of ZnO samples. From the SEM images of the above three ZnO samples, it can be seen that the morphology of ZnO changed with increasing malic acid concentration, where the ZnO sample prepared with 15 mmol malic acid perform the best 3D layered structure and complex mesh-like surface. Meanwhile, the nanostructure of ZnO was studied by TEM analysis. The TEM and HRRTEM of the ZnO samples prepared with 15 mmol malic acid are shown in Figure 4. The TEM image of a typical microsphere is shown in Figure 4a,b, showing the sheet-flower structure of ZnO. The fringe spacing of 0.28 nm is clearly observed in the HRTEM image (Figure 4c), corresponding well with the (100) lattice plane of the wurtzite ZnO (JCPDS Card No. 36-1451) [27].

### 3.2. Gas Sensing Properties

**Figure 5:**
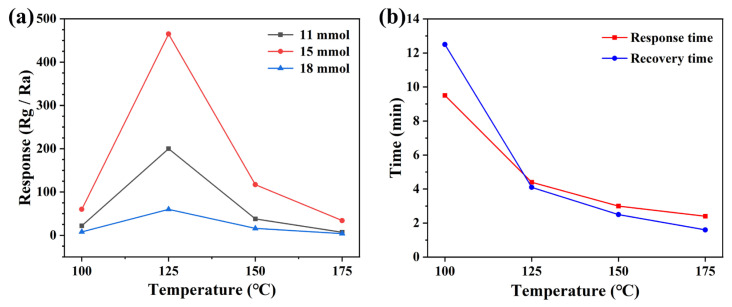
(**a**) Different sensors to 5 ppm NO_2_ vs. operating temperatures, and (**b**) response-recovery times.

It is well known that the response value of semiconductor metal oxide type gas sensors is strongly influenced by the operating temperature and therefore it is necessary to find the optimum operating temperature for gas sensors. We fabricated sensors with ZnO samples prepared from 11 mmol, 15 mmol, and 18 mmol malic acid. In order to find the optimal operating temperature, the response values of different sensors to NO_2_ at different operating temperatures were tested, and this analysis was used to obtain the optimal sensor pieces. Different sensors were studied at four operating temperatures, including 100 °C, 125 °C, 150 °C, and 175 °C, as shown in Figure 5a. From the figure, it can be seen that the response value of all three sensors fabricated for NO_2_ increases with the increase in the operating temperature, and the response value is maximum when the operating temperature reaches 125 °C. Meanwhile, the gas-sensitive response value (466.7 to 5 ppm NO_2_) of the ZnO sensor prepared based on 15 mmol malic acid to NO_2_ is much higher than the other two sensors. After that, the response values of all three sensors decreased to different degrees with a further increase in operating temperature, probably this is because the increasing operating temperature provides energy to the nitrogen dioxide to overcome the hindrance of reaction with various types of oxygen, then the content of adsorbed oxygen will increase. However, when the operating temperature exceeded the temperature of 125 °C, the adsorbed NO_2_ starts to desorb faster, resulting in a decrease in the response value of the sensor [28,29]. The response time and recovery time are also important indicators for evaluating the gas sensor. We analyzed the response and recovery times of the devices with the best response to NO_2_, and it can be seen from Figure 5b that the response time and recovery time of the sensor decrease significantly with the increase in temperature. The main reason for this phenomenon may be due to the increase in the operating temperature which makes the adsorption and desorption of gases on the surface of the sensing material faster, resulting in shorter and shorter response and recovery times [30]. In practical applications, a fast response time and recovery time are necessary.

**Figure 6:**
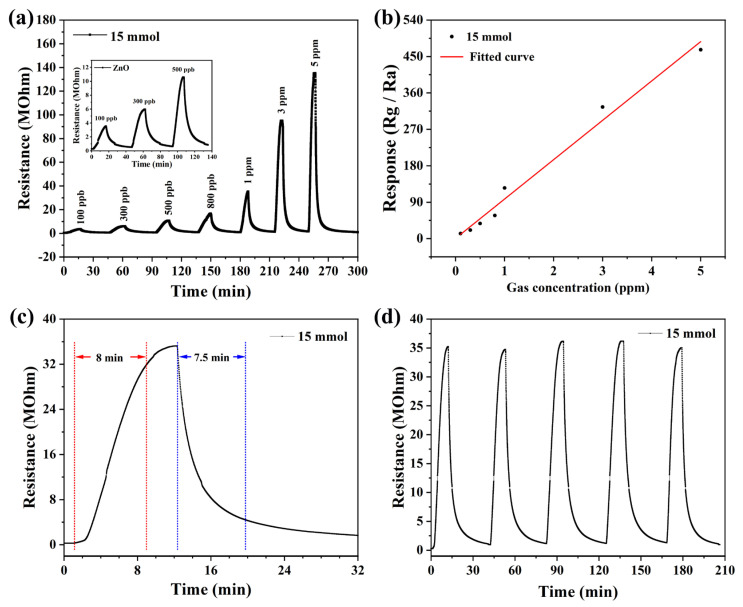
The performances of sensor to NO_2_ at 125 °C: (**a**) Transient curve, (**b**) the linear relationship of the sensor to NO_2_ in the range of 0.1 ppm to 5 ppm, (**c**) the characteristic of response–recovery curves to 1 ppm, (**d**) five periods of response curve to 1 ppm.

The response values of the three prepared devices to NO_2_ show that the ZnO gas sensor based on 15 mmol malic acid has the highest response value to NO_2_. Therefore, we continued the systematic testing of the device. Figure 6a shows the dynamic resistance curves of the sensor, exposed to different concentrations of NO_2_ at the optimal operating temperature. When the NO_2_ concentration increased from 0.1 ppm to 5 ppm, the response value also increased from 12.5 to 466.7. The sensor had a relatively high response value of 12.5 at 0.1 ppm NO_2_, which shows that the sensor has a low detection limit. In addition, it can be seen from the graph that the resistance value of the sensor increases linearly with the concentration of NO_2_, which indicates that the sensor also has good dynamic response characteristics. In addition, by fitting the response data of the sensor, the law of the response value with NO_2_ concentration can be analyzed, as shown in Figure 6b. The response value of the sensor increases linearly with the concentration of NO_2_. Figure 6c shows that the response time and recovery time are 480 s and 450 s to 1 ppm NO_2_. Additionally, repeatability is also one of the important indicators to evaluate the sensor performance. Figure 6d shows the repeatability of the sensor to 1 ppm NO_2_. From the graph, it can be reached that the response value to 1 ppm NO_2_ and the baseline resistance of the sensor is stable in five consecutive tests. The results show that the sensor has good characteristics of response/recovery and repeatability.

**Figure 7:**
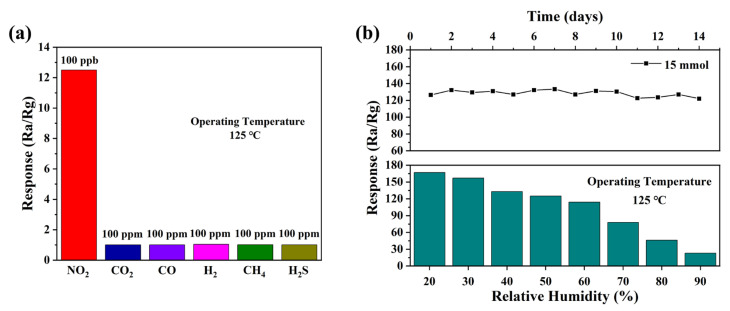
(**a**) The selectivity of sensor to 1 ppm NO_2_ and 100 ppm other gases at different temperatures, (**b**) long-term stability of the sensor at 125 °C for 1 ppm NO_2_ and response curve with relative humidity.

In addition to response and repeatability, selectivity and stability are also essential for gas sensors. Figure 7 shows the selectivity and stability of the ZnO sensor based on 15 mmol malic acid prepared for nitrogen dioxide. As shown in Figure 7a, the gas sensor based on sheet-flower ZnO performs excellent selectivity. The response value to 100 ppb NO_2_ is up to 125 but for other gases, the response values are 1.01, 1.01, 1.04, 1.02, and 1.01 to 100 ppm of CO_2_, CO, H_2_, CH_4_, and H_2_S, correspondingly. The long-term stability of the sensor to 1 ppm NO_2_ at 125 °C is shown in Figure 7b for 2 weeks. The response value of the sensor fluctuates at a constant, which shows that the sensor has stable gas-sensing performance. In addition, Figure 7b also shows the variation of the sensor response values with humidity for 1 ppm NO_2_ at 125 °C. It can be seen from the figure that the response value of the sensor to NO_2_ continues to decrease as the humidity rises. The decrease in the response value may be due to the adsorption sites on the surface of the sensing material being occupied by water molecules, reducing the adsorption of oxygen [31]. Table 1 summarizes the main results of this paper in combination with the latest research. From these results, it can be clearly seen that the gas sensor based on ZnO prepared in this paper exhibits a much more significant gas response value than other studies in gas-sensing performance and lower operating temperatures.

### 3.3. Sensing Mechanism

**Figure 8:**
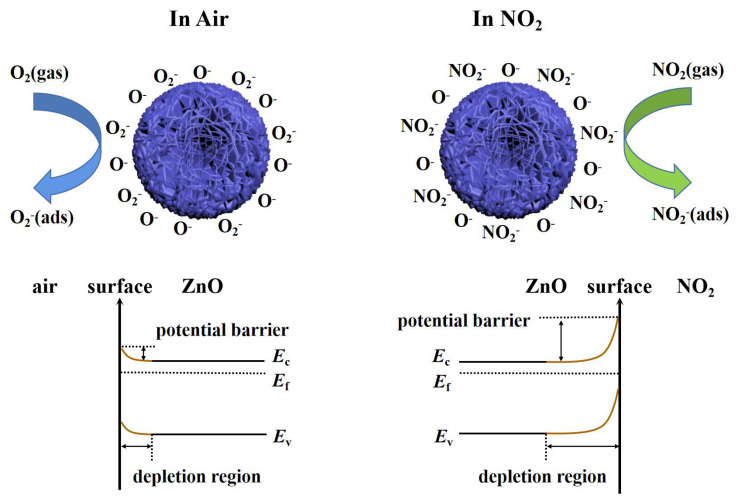
Sensing mechanism of the ZnO nanosheet−flower gas sensor.

For semiconductor gas sensors, gas-sensing performance is attributed to the interaction between gas atoms and sensing materials, which leads to changes in the resistance of the sensor. The sensing mechanism of the sensor is shown in Figure 8. Specifically, oxygen molecules are adsorbed on the surface of sheet-flower ZnO as electron acceptors in the air (Equation (1)) [39]. By capturing electrons in the conduction band of ZnO, oxygen molecules are ionized on the surface of the material to form oxygen ions. The specific type of oxygen ion formed depends on the operating temperature of the sensor. The type of oxygen ions adsorbed on the surface of the sensing material can be expressed by the following equation (Equations (2)–(4)) [40]. Therefore, an electron-depleted space charge layer is formed on the surface of ZnO, and a barrier is formed due to the decrease in carrier concentration because ZnO is an n-type semiconductor, and most carriers in such n-type semiconductors are electrons [41], which leads to the decrease in electron transport efficiency and increases the resistance of the gas-sensing material [42]. This is also known as the reference resistance in the air.

(1) $$O_{2}\;(\mathrm{gas})\;\to \;O_{2}\;(\mathrm{ads})$$

(2) $$O_{2}\;(\mathrm{ads})+e^{-}\;\to \;{O_{2}}^{-}\;(\mathrm{ads}),\;T\;<\;100\;{}^{\circ}C$$

(3) $${O_{2}}^{-}\;(\mathrm{ads})+e^{-}\;\to \;2O^{-}\;(\mathrm{ads}),\;100\;{}^{\circ}C\;<\;T\;<\;300\;{}^{\circ}C$$

(4) $$O^{-}\;(\mathrm{ads})+e^{-}\;\to \;O^{2-}\;(\mathrm{ads}),\;T\;>\;300\;{}^{\circ}C$$

In this work, the developed ZnO-based gas sensor has a lower operating temperature (T < 200 °C). When the sensor is exposed to a NO_2_ atmosphere, NO_2_ gas molecules will not only capture the electrons in the conduction band of ZnO but also interact with the oxygen species adsorbed on the surface of ZnO, electrons will be transferred from the conduction band of ZnO to NO_2_, resulting in an increase in the electron depletion layer and an increase in the resistance of the sensor (Equations (5)–(7)) [43,44]. When the sensor is again exposed to air, NO_2_ will react with holes, and electrons will be released back into the conduction band, reducing the resistance of the sensor (Equation (8)) [45].

(5) $${\mathrm{NO}}_{2}\;(\mathrm{gas})+e^{-}\;\to \;{\mathrm{NO}}_{2}^{-}\left(\mathrm{ads}\right)$$

(6) $${\mathrm{NO}}_{2}^{-}\;(\mathrm{ads})\;+\;O_{2}^{-}\;(\mathrm{ads})\;+\;e^{-}\;\to \;{2O}_{2}^{-}\;(\mathrm{ads})\;+\;{\mathrm{NO}}_{2}^{-}\;(\mathrm{ads}),\;T\;<\;100\;{}^{\circ}C$$

(7) $${\mathrm{NO}}_{2}^{-}\;(\mathrm{ads})\;+\;O^{-}\;(\mathrm{ads})+2e^{-}\;\to \;2O^{2-}\;(\mathrm{ads})+\mathrm{NO}\;(\mathrm{gas}),\;100\;{}^{\circ}C<\;T\;<300\;{}^{\circ}C$$

(8) $${\mathrm{NO}}_{2}^{-}\;(\mathrm{ads})\;+\;h^{+}\;\to \;{\mathrm{NO}}_{2}\;(\mathrm{gas})$$

Additionally, since the electron affinity (2.28 eV) of NO_2_ is higher than that of pre-adsorbed oxygen (0.43 eV) [46], NO_2_ is more easily adsorbed on the sensor surface than other gases. At the same time, the N in NO_2_ will retain an unpaired electron after forming a covalent bond between N and O [47]. The unpaired electrons from N combine with the oxygen ions on the ZnO surface, which promote chemical adsorption and improve the selectivity to NO_2_ [48].

## 4. Conclusions

In summary, sheet-flower ZnO was synthesized by a simple water bath method. The use of malic acid is feasible and beneficial to control the morphology and hierarchical structure of ZnO. The morphology and structural characteristics of the prepared materials were determined by different characterization methods. The gas-sensing of the sensor was studied by preparing the gas sensor. The results show that the sensor has a high response value to NO_2_ at a lower operating temperature (125 °C), and the response value to 1 ppm NO_2_ is 125. In addition, the sensor also has a low detection limit (100 ppb), good selectivity, and good stability. This work provides a convenient strategy for the controllable synthesis of gas-sensitive materials by a water bath method and has potential application prospects.

## Figures and Tables

**Table 1:** Comparison of this present work with reported ZnO−based NO_2_ sensors.

Material	Method	Concentration (ppm)	Operating Temperature (°C)	Response	References
ZnO	Hydrothermal	2	200	11.56	[32]
ZnO	Hydrothermal	1	200	15.3	[33]
Au/ZnO	Hydrothermal	1	150	31.4	[34]
Ag/ZnO	Hydrothermal	10	180	11.75	[35]
ZIF-8/ZnO	Liquid phase	1	200	51.41	[36]
SnO_2_/ZnO	Hydrothermal	10	150	108	[37]
ZnO-Ti_3_C_2_Tx	Hydrothermal	8	160	3.6	[38]
ZnO	Water bath	1	125	125	This work

## Data Availability

Data will be made available on reasonable request.
